# Automated detection of quiet eye durations in archery using electrooculography and comparative deep learning models

**DOI:** 10.1186/s13102-025-01284-2

**Published:** 2025-08-09

**Authors:** Fatma Söğüt, Hüseyin Yanık, Evren Değirmenci, İnci Kesilmiş, Ülkü Çömelekoğlu

**Affiliations:** 1 Vocational School of Health Service, Mersin University, Mersin, Turkey; 2 Information Systems and Technologies, Mersin University, Mersin, Turkey; 3 Department of Electrical and Electronics Engineering, Faculty of Engineering, Mersin University, Mersin, Turkey; 4 Department of Coaching Education, Faculty of Sports Science, Mersin University, Mersin, Turkey; 5 Department of Biophysics, Faculty of Medicine, Mersin University, Mersin, Turkey

**Keywords:** Quiet eye, Electrooculography, Wavelet transform, Convolutional neural networks, Long-short term memory, Transformer, UNet, GRU

## Abstract

This study presents a deep learning-based approach for the automated detection of Quiet Eye (QE) durations from electrooculography (EOG) signals in archery. QE—the final fixation or tracking of the gaze before executing a motor action—is a critical factor in precision sports. Traditional detection methods, which rely on expert evaluations, are inherently subjective, time-consuming, and inconsistent. To overcome these limitations, EOG data were collected from 10 licensed archers during controlled shooting sessions and preprocessed using a wavelet transform and a Butterworth bandpass filter for noise reduction. We implemented and compared a traditional model (SVM) and five deep learning models—CNN + LSTM, CNN + GRU, Transformer, UNet, and 1D CNN—for QE detection. The CNN + LSTM model achieved the highest accuracy (95%), followed closely by CNN + GRU (93%), demonstrating superior performance in capturing both spatial and temporal dependencies in the EOG signals. Although Transformer-based and UNet models performed competitively, they exhibited lower precision in distinguishing QE periods. The performance of the traditional model was inferior to deep learning approaches. These results indicate that deep learning provides an effective and scalable solution for objective QE analysis, substantially reducing the dependence on expert annotations. This automated approach can enhance sports training by offering real-time, data-driven feedback to athletes and coaches. Furthermore, the methodology holds promise for broader applications in cognitive and motor skill assessments across various domains. Future work will focus on expanding the dataset, enabling real-time deployment, and evaluating model generalizability across different skill levels and sports disciplines.

## Introduction

The QE is a concept explored extensively in sports psychology. It refers to the final moment of focused gaze or tracking immediately before performing a physical action. QE has been identified as a crucial predictor of a successful performance, notably in precision-based sports such as archery, golf and shooting [18]. Technically, QE is defined as the last visual fixation at least 100 milliseconds before initiating a goal-directed movement [5]. This tracking or fixation behaviour has been associated with improved sensorimotor coordination and superior performance outcomes. Fixation is defined as the focus of gaze on a stationary target, whereas tracking is defined as gaze following a moving object [10]. The QE process occurs with minimal eye movement deviations (usually between 1°-3°) and can last between 300 and 5000 milliseconds [13]. Several studies have shown that a longer duration of QE immediately before movement execution is positively correlated with improved performance on various motor tasks [6, 21]. Furthermore, QE training interventions have shown promising results in increasing athletes’ ability to regulate visual attention, especially in shooting sports such as archery [14].

The role of QE varies based on the type of motor skills required in different sports. QE is generally categorized into three groups based on skill demands: intervention skills, such as those in volleyball and ice hockey; target skills, like archery and golf; and defensive skills such as those in soccer and basketball [20]. In sports involving interference, athletes rely on the QE to anticipate and respond to incoming objects, while in target sports, QE helps improve aim and accuracy during execution. Defensive skills, on the other hand, require athletes to visually track both opponents and game dynamics, requiring flexible visual attention strategies. Importantly, while a longer QE duration is generally beneficial for target sports, it may not always be advantageous in intervention or defensive sports that require rapid decision-making and reflexive responses [15, 22].

Beyond its importance in sport performance, QE has also been associated with perceptual-motor expertise. Mann et al. [13] proposed that prolonged QE duration, together with fixation location and frequency, serves as an indicator of knowledge in sport. Studies have shown that elite athletes exhibit longer QE durations than novices [6]; this difference is evident not only between skill levels but also between successful and unsuccessful attempts of an individual athlete [19]. In archery in particular, where visual focus and stability are crucial, the ability to effectively direct visual attention in a complex field of view is critical to optimize performance [8].

To measure QE duration, researchers have used various recording techniques as an alternative to traditional mobile eye-tracking devices. EOG offers advantages in terms of timing resolution when measuring eye movements based on the cornea-retina potential difference. Human eye movements are usually accelerated up to 100 Hz, which requires sampling rates of at least 200 Hz to avoid signal distortion, according to the Nyquist-Shannon theory [16]. However, conventional mobile eye-tracking systems operate at only 30 Hz, which may be insufficient to capture fast, dynamic visual behaviors [4]. EOG with higher timing resolution makes it possible to precisely analyze eye movement patterns and QE durations [8]. Despite these advantages, traditional QE detection methods have relied on manual expert annotation using EOG, which is subjective, labor-intensive and prone to variability.

Recently, advances in machine learning (ML) and deep learning (DL) have enabled increased accuracy and efficiency in automated QE detection. Traditional ML approaches are methods such as Support Vector Machines (SVM) and Random Forests applied to time-series EOG data; however, these models are highly dependent on manually extracted features, which limits their ability to capture complex temporal dependencies [24]. Deep learning methods, particularly Convolutional Neural Networks (CNNs), have proven to be highly effective at identifying spatial features directly from raw EOG data, eliminating the need for manually designed features [2]. However, since detecting QE involves analyzing patterns that change over time, Recurrent Neural Networks (RNNs), like Long Short-Term Memory (LSTM) networks, are well-suited for this task as they can accurately capture and model long-term temporal relationships.

To exploit the strengths of CNNs and LSTMs, hybrid architectures such as CNN + LSTM and CNN + GRU (Gated Recurrent Units) have been proposed. By integrating CNNs for spatial feature extraction and LSTMs or GRUs for temporal sequence modelling, these models become particularly effective for EOG-based QE detection. Besides recurrent networks, Transformer models using self-attention mechanisms have shown promising results in processing long-range dependencies in sequential data. Furthermore, UNet-based architectures, initially developed for image segmentation, can be adapted to detect QE regions in time-series EOG data. Finally, 1D CNNs stand out as a computationally efficient alternative offering fast and lightweight feature extraction capabilities.

### Current study

This study proposes a novel deep learning-based framework for automatic QE detection using EOG signals, addressing the limitations of expert-based annotation. Unlike prior research, which primarily relied on manual methods or traditional machine learning, we employ multiple state-of-the-art deep learning architectures, including:

- 1D CNN: A lightweight architecture optimized for time-series analysis.
- Transformer: Utilizes self-attention mechanisms for sequence modelling.
- UNet: Adapts segmentation-based learning for QE detection.
- CNN + GRU: Similar to LSTM but with reduced computational complexity.
- CNN + LSTM: Captures both spatial and temporal dependencies.

EOG data were recorded from 10 experienced archers during controlled shooting sessions using a mobile EOG device. Signals were pre-processed using wavelet transform and a Butterworth filter to remove noise. Expert-annotated QE durations served as ground truth labels for training the models. Data augmentation techniques, including time shifting and Gaussian noise addition, were applied to enhance model generalization.

The objective of this study is to determine the most accurate and efficient deep learning model for QE detection in archers, assess whether deep learning models can be effectively applied for QE detection and to evaluate its implications for sports performance analysis. Our preliminary findings demonstrate that deep learning can provide a scalable, objective alternative to traditional expert-based QE measurement, with the CNN + LSTM model achieving up to 95% accuracy. This research represents a significant step towards deep learning-based QE detection in archery, laying a potential foundation for future real-time feedback systems and advanced training methodologies in sports psychology and motor control research. Although real-time performance metrics were not directly evaluated in the current study, the high accuracy rates achieved indicate the potential in this direction.

## Materials and methods

### Participants and data collection

This study was conducted with 10 licensed archers from the Archery Sports Club in Mersin, Turkey. All participants have been actively training for at least two years. The average height of the participants was 160.93 ± 11.33 cm and the average body weight was 63.67 ± 19.34 kg. The athletes trained four days a week for two hours each time under standardized training conditions.

To maintain measurement accuracy and participant focus, only researchers and the archer being measured were present in the experimental area, with all necessary safety precautions supervised by a professional trainer.

### Eye movement recordings and QE duration

**Fig. 1:**
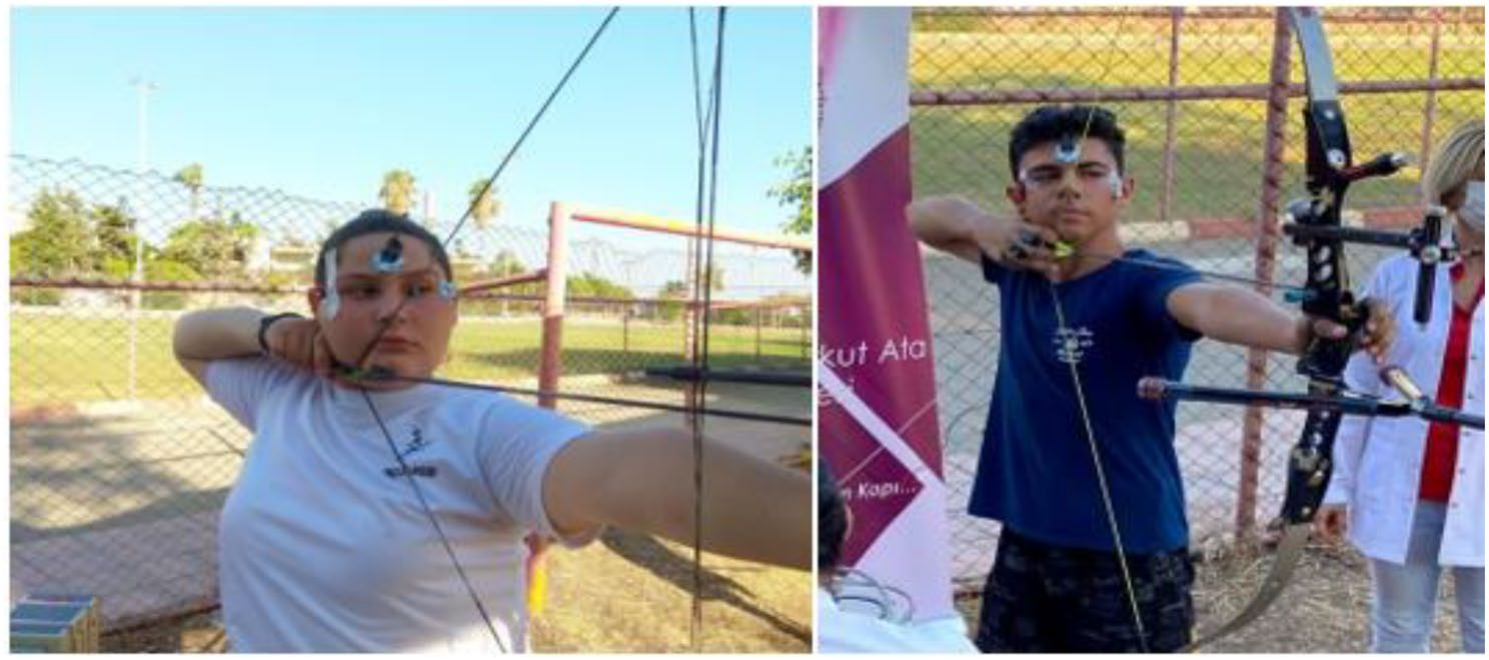
Electrode placement and archers’ stance during QE measurements (left) horizontal EOG recording (right) vertical EOG recording

Eye movements were recorded using EOG, a technique that captures eye movement dynamics based on the cornea-retina potential difference. Disc-shaped Ag/AgCl electrodes were used for data acquisition, with electrode placements ensuring reliable detection of both horizontal and vertical eye movements (Fig. 1). Data were collected simultaneously with each arrow release using a BIOPAC MP100 electrophysiological recording system (BIOPAC Systems, Inc., Santa Barbara, CA, USA).

Given the single-channel nature of the amplifier, horizontal and vertical recordings were conducted separately. The EOG signals were sampled at 200 Hz, consistent with the Nyquist-Shannon theorem for capturing rapid eye movement fluctuations [16]. Initial signal analysis was performed using BIOPAC Acknowledge Analysis Software (ACK 100 W). The QE duration was defined as the time elapsed from the start of gaze fixation until arrow release, with the QE end time manually annotated by an expert based on the sound of the arrow leaving the bow. A single researcher, present during each shooting session, consistently annotated the quiet eye offset time for all shots. These annotations were conducted in real time, precisely synchronized with the audible moment of arrow release from the bow.

### Signal analysis methodology and the proposed technique

For adopting the optimal input to the deep learning models, preliminary studies were performed and we evaluated three scenarios: Scenario (A) model performance without any preprocessing steps, Scenario (B) model performance with only Butterworth filtering applied, and Scenario (C) model performance with both Wavelet decomposition and Butterworth filtering applied.

**Fig. 2:**
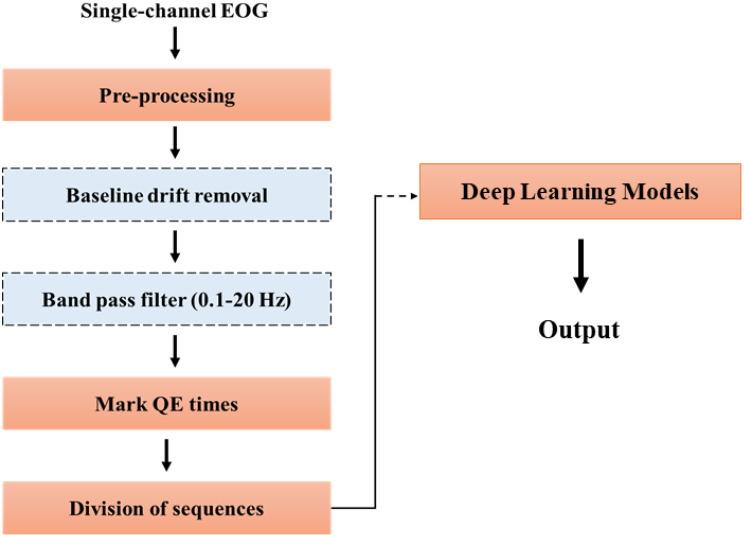
Workflow for Quiet Eye detection using EOG data and deep learning models

The general schematic representation of the proposed approach is illustrated in Fig. 2. Briefly; after obtaining the data from archers using single channel EOG, pre-processing was applied to data to filter out baseline drift noises and unwanted parts. Then, marked QE times by experts are applied to the signals to create QE sequences from obtained signals. These sequences are fed into deep learning models to predict QE times automatically after a shooting section. Detailed explanation of the methods is given below.

### Data pre-processing

To ensure high signal quality, raw EOG signals underwent preprocessing to remove artifacts and baseline drift noise. The wavelet transform method was employed to filter out low-frequency fluctuations, using the following equation:

1 $$\:W\:f\left(a,b\right)=\:{\int\:}_{-\infty\:}^{\infty\:}f\left(t\right){\psi\:}_{a,b}^{*}\left(t\right)dt$$

**Fig. 3:**
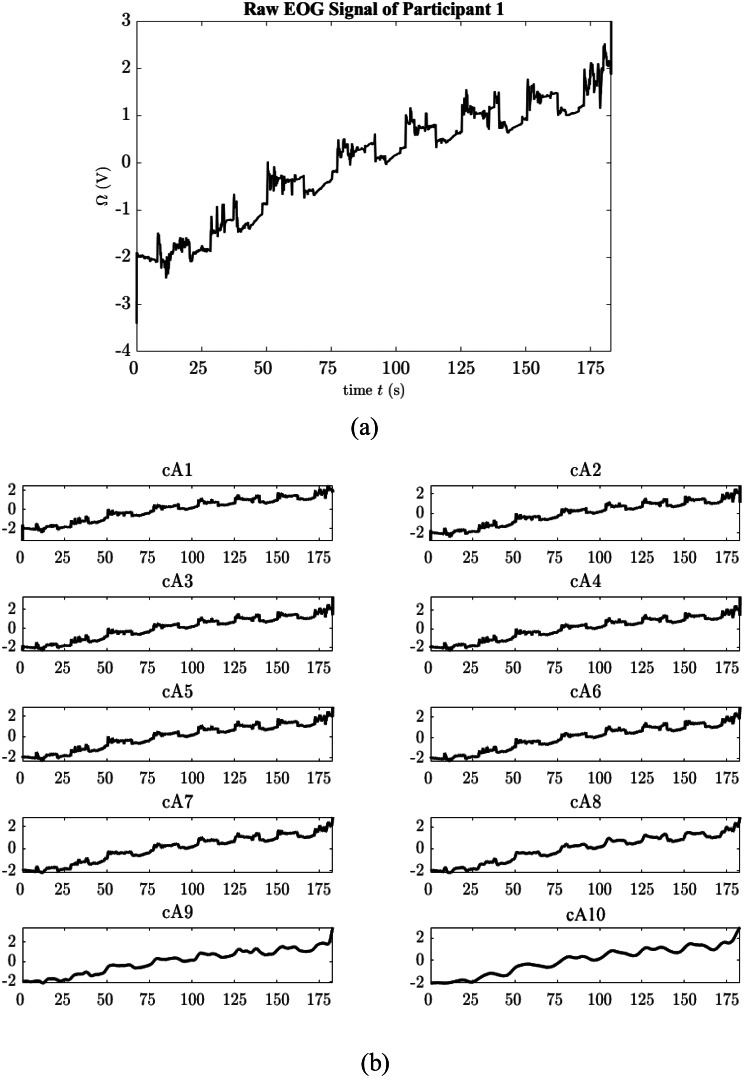
**a**) Raw EOG data **b**) Approximation coefficients of the raw EOG signal after 10 level wavelet decomposition

Here ψ (t) denotes the wavelet function and in the equation, complex conjugate of the function is used since it only responds to the nonnegative frequencies [12]. Wavelet transform provides time-frequency resolution in different frequency bands using basic functions called mother wavelets. ψ(a, b) is called the mother wavelet and a and b represent the scale and translation parameters. Selection of mother wavelet in the function is an important task and mother wavelet chosen based on similarity with the main signal. Since our signals are discrete, we used wavelet decomposition method to evaluate different frequency bands. Using this method, the signals are separated into sub bands and the relevant time frequency components are obtained. These sub-signals include approximation and detail coefficients, which correspond to the low and high frequency components of the signal, respectively. In an N-point decomposition, the signal is decomposed into N details, 1 approximation coefficient, and the expression of the signal is as follows [23]:

2 $$\:f\left(x\right)\:=\:c{A}_{1}+\:c{D}_{1}+\:c{D}_{2}\:+\:\dots\:\:+\:c{D}_{N}$$

where cA_1_ is the approximate coefficient, while cDn shows the detail coefficients. Here n shows the level of decomposition. Figure 3a shows a raw EOG signal recorded from an individual during six-shot experiment. This signal has been subjected to 10 levels of decomposition and the approximation coefficients from this decomposition are given in the Fig. 3b.

**Fig. 4:**
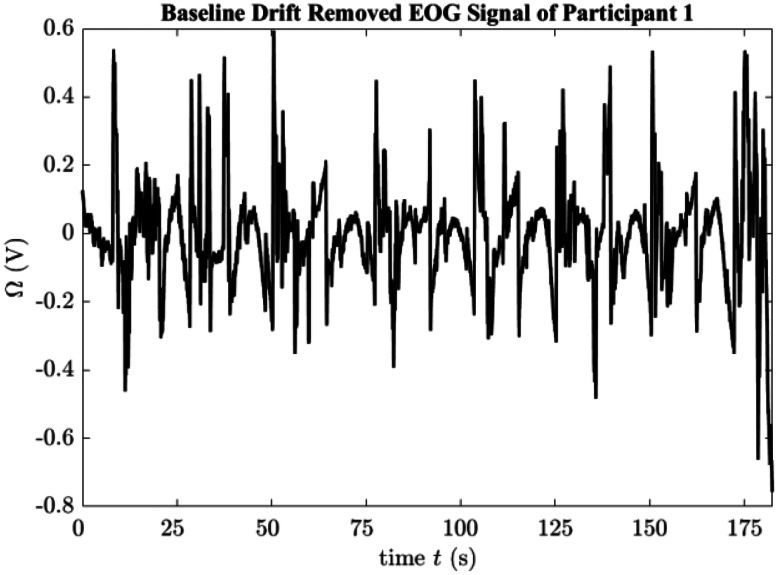
Baseline drift removed EOG signal or Participant 1 over time (t)

As can be seen from the Figs. 3b and 10th approximation coefficient directly corresponds to the baseline of the raw signal. Other approximation coefficients still contain useful information about the signal, therefore parsing should be done carefully. When the cA_10_ coefficient was subtracted from the main signal, baseline drift removed signal is obtained as seen in Fig. 4.

**Fig. 5:**
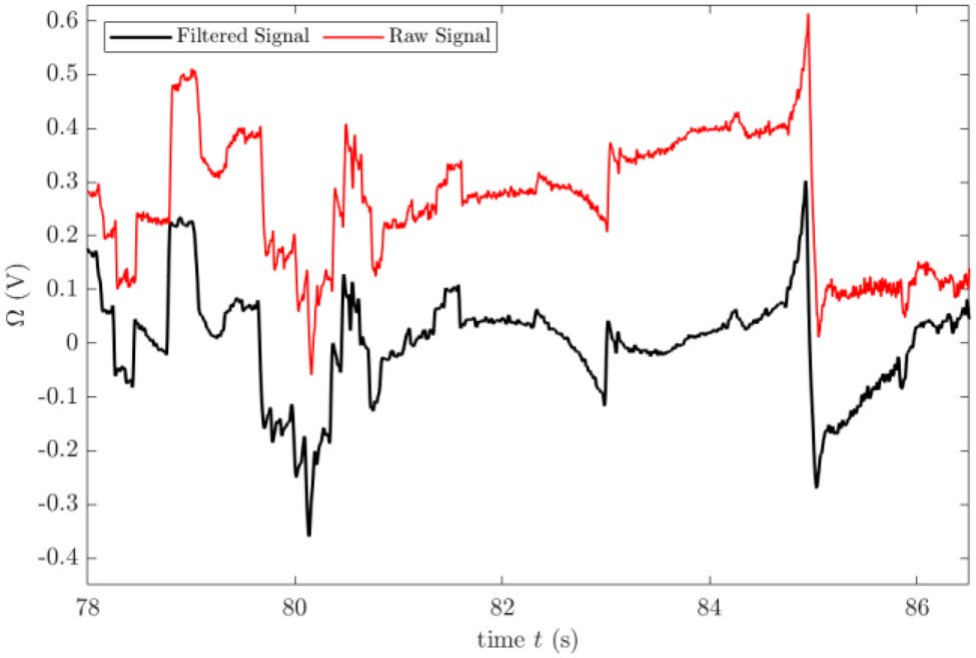
Comparison of raw and filtered EOG signals over time (t) between 78 and 86 s

The frequency range of EOG signals lies between 0.1 and 20 Hz [3]. In order to filter out the noise components in this range, a band pass Butterworth filter is designed and the signal is de-noised using this filter. Figure 5 shows the filtered and raw signals in the same graph.

**Fig. 6:**
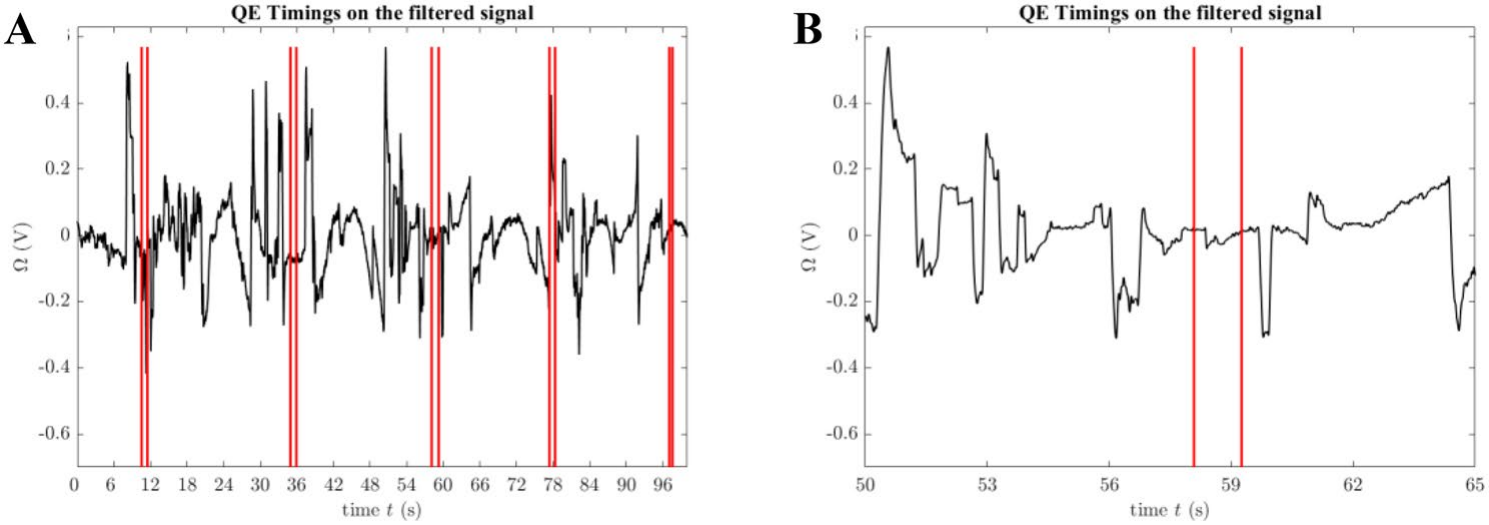
QE timings on the filtered EOG signal. (**A**) Full signal with QE timings highlighted in red. (**B**) Zoomed-in view of the signal between 50 and 65 s, showing detailed QE timing intervals

The QE points, noted by an expert during the arrow shooting, were indicated on the signal as in Fig. 6A, and some zoomed part in Fig. 6B.

### Data preparation and augmentation

In order to implement the proposed deep learning-based QE detection technique, the EOG signals were segmented around the QE duration. Specifically, for each arrow shot, the recorded signal was trimmed from 2.5 s before to 6 s after the QE duration, resulting in 1700-sample-long sequences. This process was applied to 10 participants, yielding a dataset of 60 labelled QE-containing signals.

To detect Quiet Eye (QE) periods from EOG signals, segmented EOG signals were provided as input to the trained model. Consequently, the model output consisted of a signal composed of sequences of 0 (non-QE periods) and 1 (QE periods). This signal was chronologically scanned to identify transitions between the two states: a transition from 0 to 1 defined the QE onset event at time ton_pred (predicted onset), while a transition from 1 to 0 indicated the QE offset event at time toff_pred (predicted offset). These predicted events were compared to the ground-truth onset (ton) and offset (toff) times. This process was carried out as follows: a time tolerance (T) of 100 ms was adopted, as suggested in [7]. Subsequently, a predicted event at time ton_pred or toff_pred was acknowledged as a true positive if an event of the same type occurred in the ground-truth signal at time ton or toff, such that ton - ton_pred < T or toff - toff_pred < T. Otherwise, the predicted event was classified as a false positive. Additionally, error metrics were calculated to evaluate the temporal accuracy of predictions, including Bias_on = ton_pred - ton for onset events and Bias_off = toff_pred - toff for offset events.

To ensure robust model generalization and prevent overfitting, data augmentation techniques were applied to the extracted signals. The augmentation methods included:

- Time shifting: Slight temporal displacements were introduced to simulate natural variations in gaze fixation patterns.
- Gaussian noise addition: Low-intensity Gaussian noise was injected into the signals to enhance model robustness against minor signal fluctuations.

Additionally, for training purposes, a binary dataset was generated where:

- Ones (1s) represented QE durations, corresponding to periods of gaze fixation.
- Zeros (0s) denoted non-QE periods, corresponding to other phases of the signal.

**Fig. 7:**
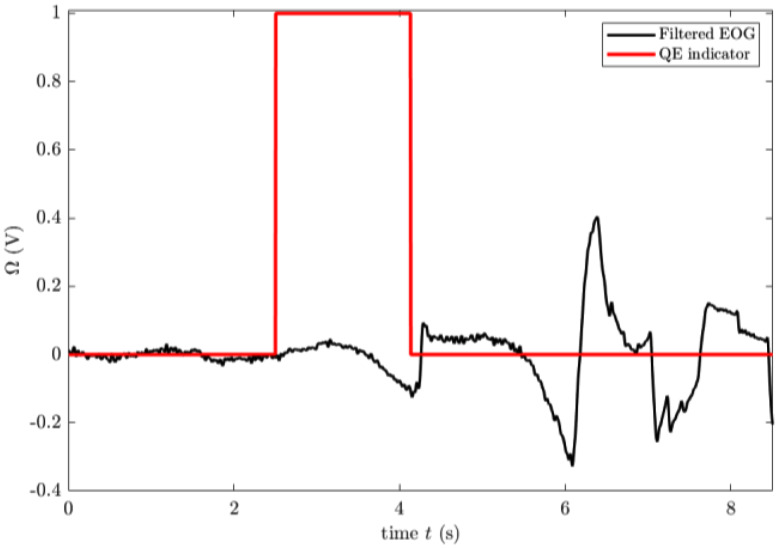
Visualization of the filtered EOG signal (black) with the corresponding QE indicator (red) showcasing binary labeling over time (t)

This pre-processing step allowed the deep learning models to learn the temporal structure of QE events more effectively. Figure 7 illustrates a sample visualization of the extracted QE signal with its corresponding binary labelling.

In order to evaluate the performance of the model and to avoid data leakage, the dataset is systematically divided into training, validation and testing phases. Considering the small sample size, the 5-fold cross-validation method was adopted to obtain more reliable and unbiased results. In this approach, the data set was divided into five equal parts, four parts were used as training and one part as test set in each fold.

### Support vector machine

Support Vector Machine (SVM) is a powerful supervised learning algorithm used in machine learning for classification and regression problems. SVM works by constructing a hyperplane of data points in a way that provides the best separation between classes; this hyperplane focuses on maximizing the margin (the distance to the nearest data points) [9]. In particular, for data that is not linearly separable, kernel functions (e.g. RBF kernel) are used to move the data into a higher dimensional space where it can be discriminated. In our Quiet Eye (QE) detection study, SVM was used as the conventional method to detect QE and non-QE periods from EOG signals, which will be compared with deep learning approaches. For this purpose, the signal data were normalized, standard scaling was applied and each time point was treated as a separate sample. The SVM model configured with “RBF kernel” is evaluated with performance metrics such as accuracy, precision, recall and F1 score.

### Deep learning architectures

To automatically detect the QE durations from EOG signals, we implemented and evaluated multiple deep learning architectures, focusing on both spatial and temporal feature extraction. In particular, we propose two hybrid models: CNN-LSTM and CNN-GRU, which combine CNN for feature extraction with recurrent layers for sequential modelling. These models were compared against three additional architectures: Transformer, UNet, and CNN-only models, each offering distinct advantages in time-series signal analysis. All models were trained for 50 epochs using the “Adam optimizer” (learning rate = 0.001) with a “Binary_crossentropy” loss function.

Adam (Adaptive Moment Estimation) was chosen as the optimizer because this algorithm is known for achieving fast and stable convergence in deep learning models. By combining momentum and RMSprop methods, Adam adaptively adjusts the learning rate, which speeds up the training process, especially in models with complex architectures (e.g., hybrid structures such as CNN-LSTM). In the QE detection problem, given the time-series nature of the EOG signals and the multi-layer structure of the model, the Adam optimizer reduces the risk of getting stuck in local minima during gradient descent and provides better generalization performance. Moreover, Adam’s low hyperparameter tuning requirement (the default learning rate is usually sufficient) offers a practical advantage in the model development process. For these reasons, the Adam optimizer was preferred for training the QE detection model.

QE detection is essentially a binary classification problem, with each time point belonging to either a QE period (1) or a non-QE period (0). “Binary_crossentropy” is a loss function commonly used in binary classification problems because it allows to optimize the probabilistic predictions of the model by measuring the difference between the model’s outputs and the true labels. This function helps the model to learn both classes correctly, especially in cases where there are class imbalances (e.g. QE periods are less frequent than non-QE periods). Furthermore, binary_crossentropy, when used in combination with the sigmoid activation function, makes it possible to interpret the outputs of the model as a probability between 0 and 1, which is suitable for thresholding operations in the detection of QE start and end points (onset and offset). Therefore, in binary classification-based time series analysis such as QE detection, binary_crossentropy is an ideal choice to maximize the performance of the model.

Each models performance was assessed using accuracy, precision, recall, and F1-score metrics. In addition, error metrics are also evaluated for onset and offset detections.

#### CNN model

A CNN is a deep learning network structure that learns directly from data and does not require manual feature extraction [2]. CNNs are particularly useful in finding patterns to detect events. A lightweight 1D CNN model was applied to extract features from raw EOG signals. This model used two convolution layers (64 and 128 filters) with varying kernel sizes to capture different temporal resolutions. Both CNN layers used the ReLU (Rectified Linear Unit) activation function:

3 $$\:ReLU\left(x\right)=max(0,x)$$

where x is the input array to the ReLU function. ReLU function is used as it helps to prevent the exponential growth of neural network computational cost.

Max pooling layers downsample extracted features, enhancing computational efficiency. An upsampling layer restores sequence length to align with original data format. A final Conv1D layer with “sigmoid activation function” generated QE classifications.

#### Transformer model

The Transformer-based model employs self-attention mechanisms to process long-range dependencies in sequential data [1]. Unlike recurrent models, Transformers compute dependencies in parallel, enhancing efficiency while preserving global contextual relationships. It contains an initial convolutional layer with 64 filters and kernel size of 3 while using ReLU as activation function. This layer extracts low-level spatial features. “Layer normalization” stabilizes feature scaling before applying attentional mechanisms. “Multi-headed self-attention” enables dynamic weighting of temporal dependencies. Residual connections preserve information flow and prevent gradient vanishing. A final Conv1D layer produces QE classifications with a “sigmoid activation function”. We used this model to capture global dependencies in the QE sequences.

#### UNet model

The UNet architecture is widely known for its segmentation capabilities and is suitable for detecting QE durations as continuous temporal segments rather than discrete classifications [17]. It enables precise region-based learning, which is critical for identifying QE phases within longer signal sequences. We used 3 “convolution layers” (64, 128 and 256 filters) with the “ReLU activation function” to extract hierarchical feature representations. “Max pooling layers” progressively down-sample the signal to increase feature abstraction. “Up-sampling layers” are used to restore temporal resolution for accurate segmentation of QE durations. A final “Conv1D layer” is designed with a “sigmoid activation function” to generate segmentation masks for QE classification. The UNet model is developed for precise segmentation of QE events compared to classification models. It is capable of capturing both local and global spatial dependencies, improving segmentation accuracy.

#### CNN-GRU model

GRU offer an efficient alternative that preserves the ability to capture temporal dependencies using less parameters compared to LSTM networks (Cho et al., 2014). This makes GRU less computationally demanding and suitable for real-time applications [11]. In this study, we introduce a CNN-GRU model for automatic QE detection using CNN for spatial feature extraction and GRU for learning temporal dependencies in EOG signals.

The model follows a three-step process. At first, CNN layers extract low-level features from the raw EOG signal, highlighting important spatial patterns. These convolutional layers allow the model to focus on the main aspects of the signal without requiring manual feature engineering. Next, GRU layers process these extracted features to learn their evolution over time and capture long-range dependencies critical for accurate QE detection. Finally, the processed features are passed through “fully connected layers” to produce the final output, classifying each time step as part of a QE period.

Compared to the CNN-LSTM model, the CNN-GRU model achieves similar accuracy while reducing computational complexity. GRU’s simplified gating mechanism enables faster training and inference while maintaining strong temporal modeling capabilities. This makes the CNN-GRU approach a promising option for real-time Quiet Eye analysis in sports and cognitive research.

#### CNN-LSTM model

**Fig. 8:**
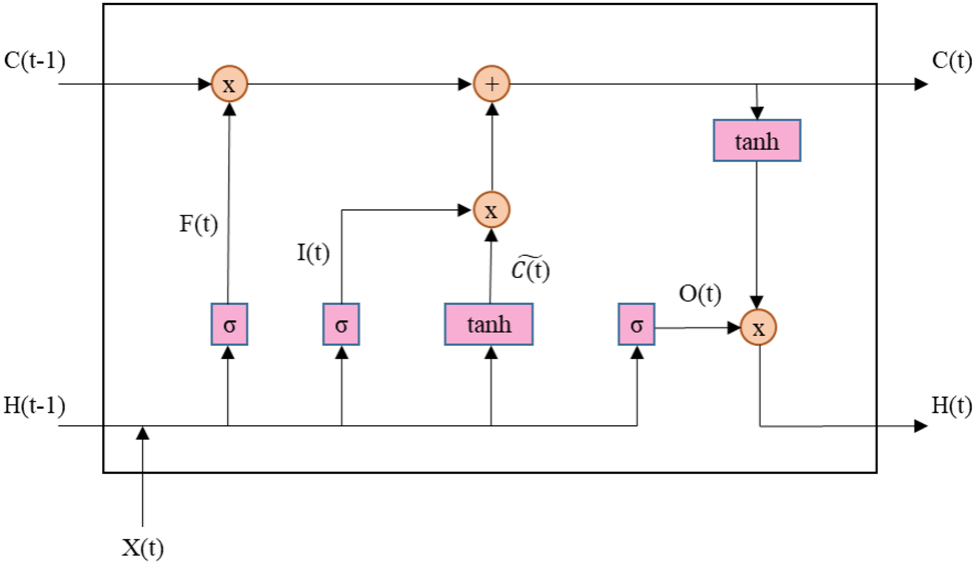
Diagram of a LSTM cell showcasing its internal operations, including forget gate F(t), input gate I(t), cell state update $\:\stackrel{\sim}{C\left(t\right)},$ and output gate O(t). Activation functions such as sigmoid σ and hyperbolic tangent tanh are highlighted

LSTM is an artificial neural network architecture which mostly used for both prediction and classification in time-series [24]. Unlike traditional neural networks, LSTM uses feedback connections and could process both instant data and data arrays. Compared to traditional RNNs, LSTM networks cover a long-term memory mechanism, effectively solving the vanishing gradient problem. LSTM utilizes three key gates to adjust the information flow within the memory cell. The forget gate decides how much of the previous information should be kept, while the input gate handles how much new information is fed into the current cell state. Finally, the output gate regulates the amount of processed information passed to the next layer, ensuring effective long-term dependency learning. Figure 8 shows the basic structure of a LSTM network.

In the LSTM architecture depicted in Fig. 8, each memory cell contains three main gates—forget, input, and output—which collectively manage how information is stored, updated, and propagated. The activation functions used in these gates are the sigmoid (Eq. 10) and the hyperbolic tangent (Eq. 11). While the sigmoid function σ (⋅) constrains outputs to the range [0,1], allowing selective passage or blocking of information, the tanh (⋅) function helps alleviate vanishing gradient issues by maintaining a richer gradient flow. The following set of Eq. (4) to (11) outlines the detailed operations within one LSTM cell:

4 $$\:{f}_{t}={\upsigma\:}({W}_{f}\left[{h}_{t-1},{x}_{t}\right]+{b}_{f})\:$$

5 $$\:{i}_{t}={\upsigma\:}({W}_{i}\left[{h}_{t-1},{x}_{t}\right]+{b}_{i})\:$$

6 $$\:{\stackrel{\sim}{C}}_{t}=\text{t}\text{a}\text{n}\text{h}({W}_{c}\left[{h}_{t-1},{x}_{t}\right]+{b}_{c})\:$$

7 $$\:{C_t} = {f_t} \odot {C_{t - 1}} + {i_t}\: \odot {\mathop C\limits^ \sim _{t\:}}$$

8 $$\:{o}_{t}={\upsigma\:}({W}_{o}\left[{h}_{t-1},{x}_{t}\right]+\text{o}\:$$

9 $$\:{h_t} = {o_t} \odot {\rm{tanh}}\left({{C_t}} \right)\:$$

10 $$\:{\upsigma\:}\left(\text{x}\right)=\frac{1}{1+{e}^{-x}}\:$$

11 $$\:\text{t}\text{a}\text{n}\text{h}\left(\text{x}\right)=\frac{{e}^{x}-{e}^{-x}}{{e}^{x}+{e}^{-x}}$$

Equation 4 which corresponds to the *Forget Gate*, determines which information from previous cell state should be discarded or retained. *Input Gate* controls how much new information is added to the cell state and given by Eq. 5. *Cell States* generates and updates the cell stated by new candidate values. Equation 6 and Eq. 7 shows the *Candidate Cell State* and *Cell State Update* parameters. *Output Gate* $\:{o}_{t}$ determines how much of the updated cell state is fed into the next hidden state. Finally, *Hidden State* $\:{h}_{t}$ produces the hidden state output by applying the output gate into the updated cell state.

In these equations, $\:{x}_{t}$ is the input vector at time t, $\:{h}_{t-1}$​ and $\:{h}_{t}$​ are the hidden states at consecutive time steps, and $\:{C}_{t-1}$ and $\:{C}_{t}$ represent the cell states. The weight matrices $\:{W}_{f}$​,$\:{W}_{i}$,$\:\:{W}_{c}$ and $\:{W}_{o}$ are learned parameters that correspond to each gate, while $\:{b}_{f}$​,$\:{b}_{i}$,$\:\:{b}_{c}$ and $\:{b}_{o}$ are the respective bias terms. The operator ⊙ denotes element-wise multiplication. By dynamically regulating the flow of information through these gates, LSTM networks effectively capture both short- and long-term dependencies in sequential data.

In our model, output of the CNN layer connects into the first LSTM layer for predicting the QE durations using the features obtained from CNN layers. We used two bidirectional LSTM layers since EOG signals have similar intervals as the QE durations that make data more complex to detect.

Proposed model is trained by minimizing a loss function. Our data contains the QE duration or non-QE duration. Therefore, we used categorical cross-entropy loss function described as,

12 $$\:CE=-log\left(\frac{{e}^{{S}_{p}}}{{\sum\:}_{j=1}^{C}{e}^{{S}_{j}}}\right)$$

where $\:C$ is the probability over classes and $\:{S}_{p}\:$is the score of the positive classes. $\:{S}_{j}$ represents the logits for all $\:C$ classes. Adam optimizer is used with a learning rate of 0.001. As evaluation metrics we determined accuracy, precision, recall, and F1-Score.

**Fig. 9:**
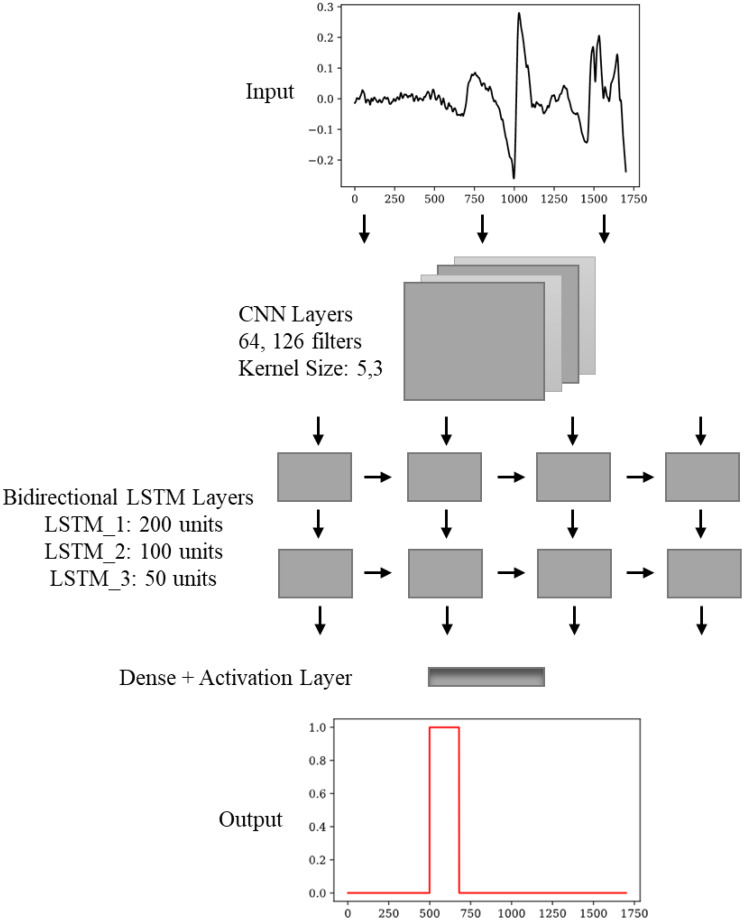
Architecture of the proposed CNN-LSTM model: The input signal is processed through convolutional layers for feature extraction, followed by LSTM layers for temporal modeling, and finally binary classification output indicating event detection

In this study, a CNN-LSTM model is proposed to provide the QE detection. The algorithm is trained to take the preprocessed data and work to reveal the pattern. The purpose of the CNN layers is to extract spatial features from the EOG signals. These extracted features are given into the LSTM layers, which predict the next QE durations using the previous ones. We used bidirectional LSTM layers for temporal dependency modelling, which effectively captures localized signal features. Since QE durations of EOG signals show repetitive patterns, CNN-LSTM performs superior prediction compared the CNN only model. The architecture of the proposed model is given in Fig. 9. The detailed explanation of the proposed model is given below the figure.

In the proposed model, we had two convolutional layers containing 64 and 128 filters with kernel sizes of 5 and 3. ReLU activation function with batch normalization is applied to extract spatial features without overfitting. Max pooling layers downsample the extracted feature maps which causes to reduce dimensionality while retaining essential information. Bidirectional LSTM layers (200, 100 and 50 units, L2 regularization = 0.001) capture forward and backward temporal dependencies in QE sequences. Upsampling layers restored the original sequence length to ensure consistent temporal structure. A fully connected dense layer with sigmoid activation function produced binary predictions for QE extraction. This model, effectively works on long-term temporal dependencies which causes to outperform CNN and other models. This models ability to capture both spatial and temporal features make it highly suitable for sequential physiological signals.

### Statistics

In order to further evaluate the performance of the models and statistically analyze the differences between different scenarios, a paired t-test was applied on the 10-fold cross-validation results. This test was used to compare the performance of the models in Accuracy, PPV (Positive Predictive Value), SEN (Sensitivity), F1-Score and MAE (Mean Absolute Error) metrics. In the Results and Discussion section, the results of the comparisons made for each scenario and metric are presented in detail.

## Results and discussion

In this study, neural network algorithms are implemented in anaconda environment with Python 3.10 using Tensorflow framework and Keras library, while the signal preprocessing algorithms are implemented in MATLAB 2023b. Hardware specifications are given in Table 1.

**Table 1:** Hardware specifications

Feature	Description
OS	Windows 10 Home 64 Bits
CPU	Intel(R) Core(TM) i7-6500U CPU @ 2.50 GHz 2.59 GHz
RAM	32,00 GB
GPU	NVIDIA Tesla T4 15,109 MiB @1590 MHz, 70 W

**Fig. 10:**
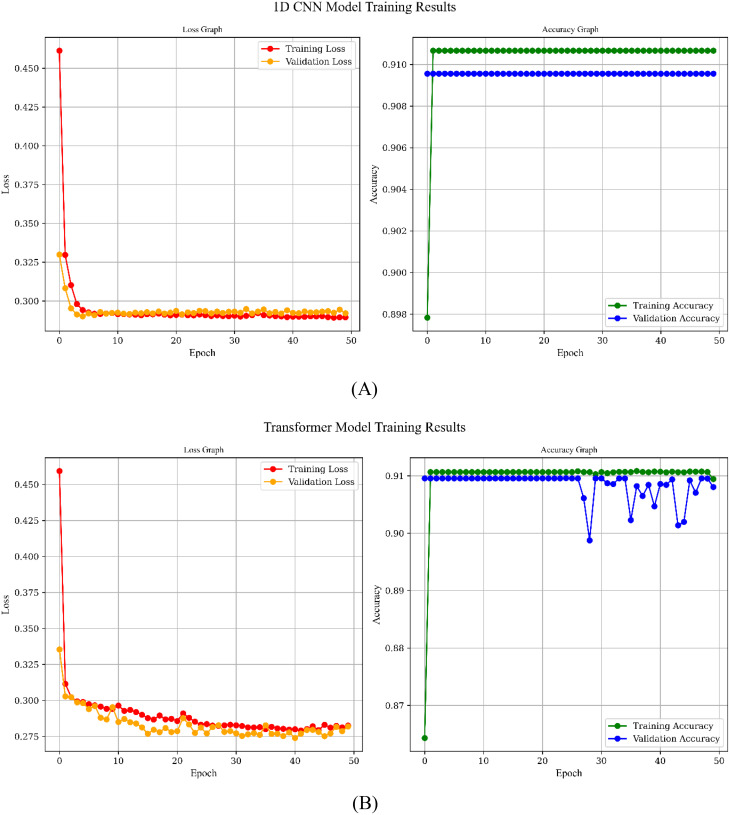

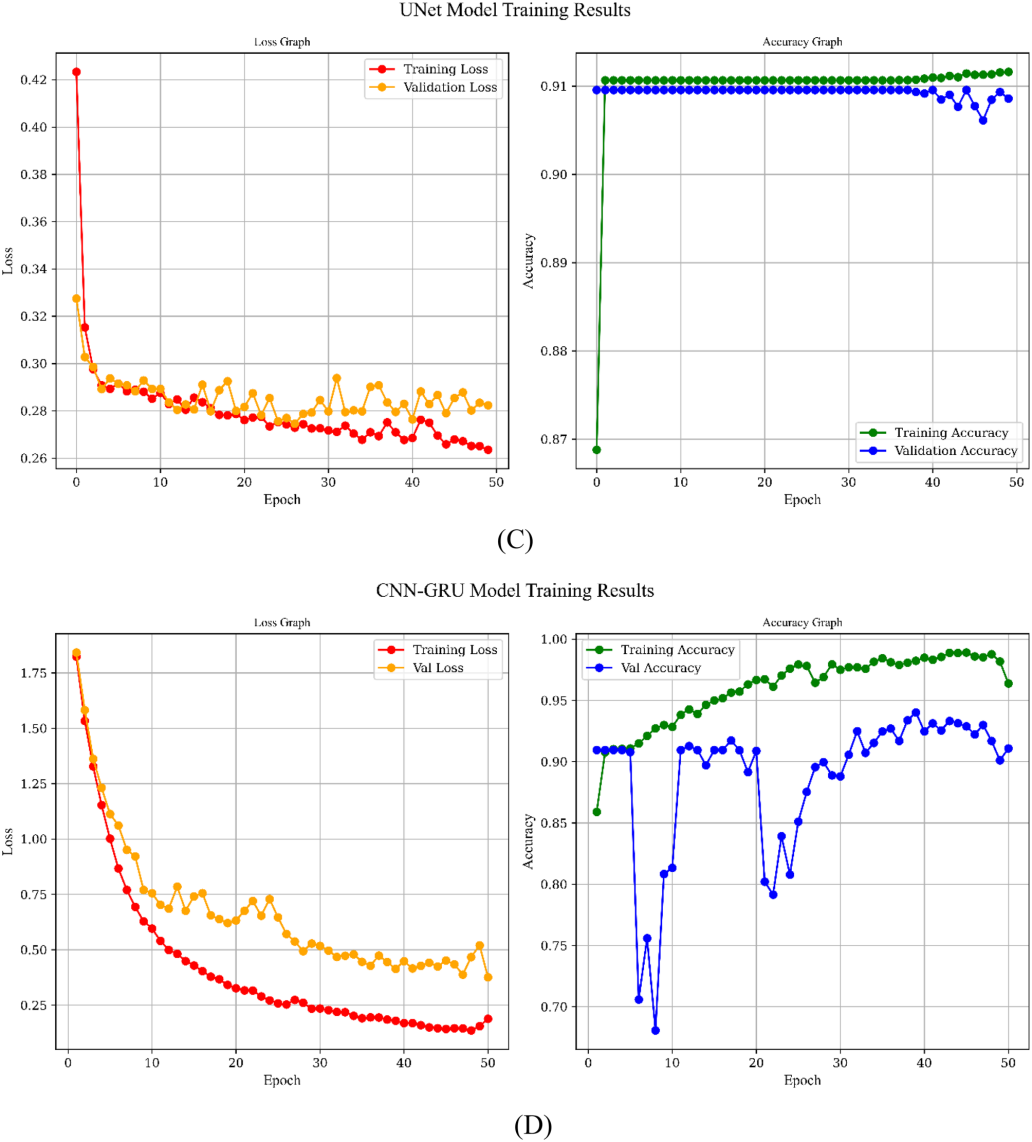

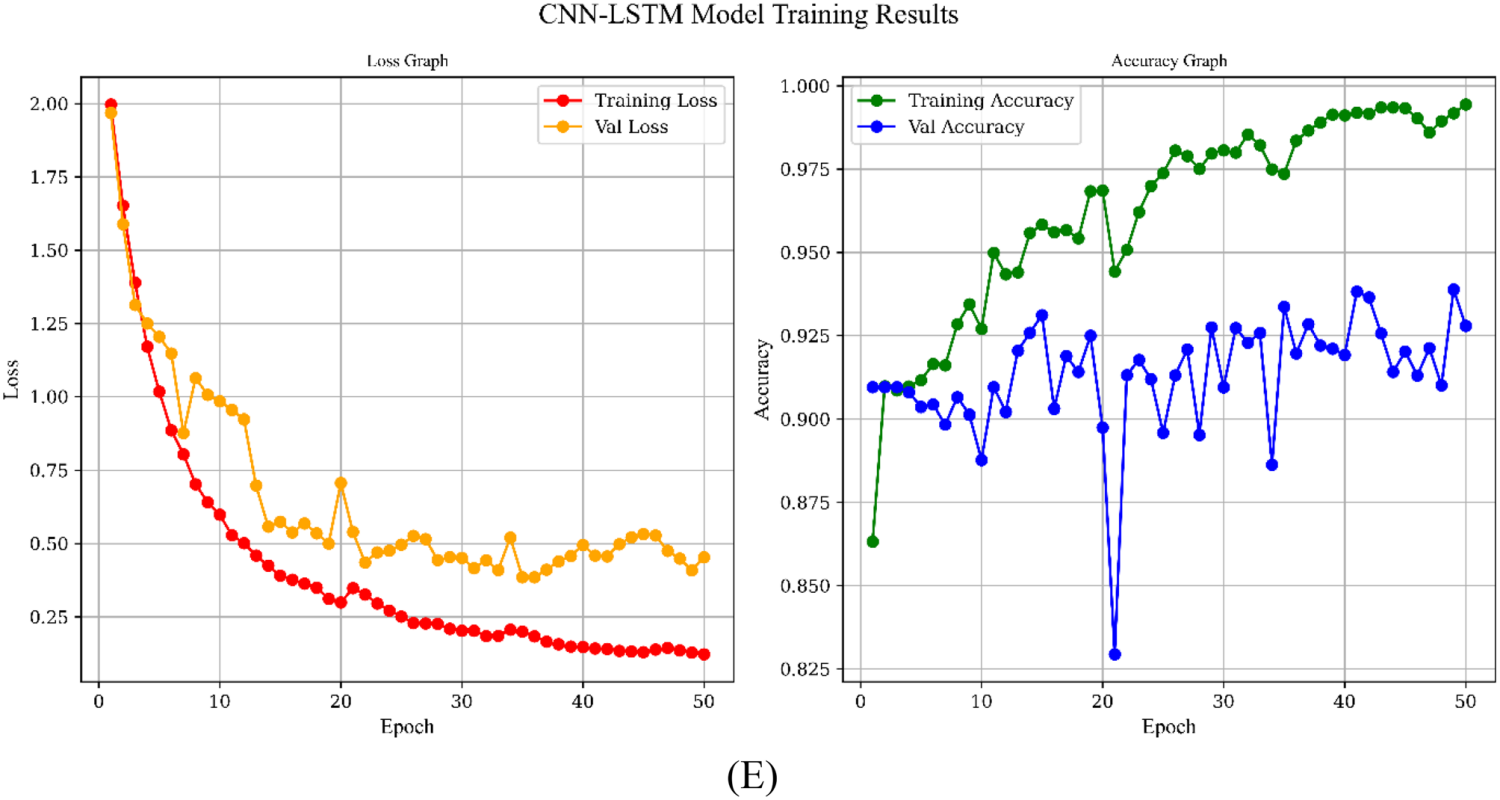
Learning Curves of the models over 50 epochs: **A**) CNN Model, **B**) Transformer Model, **C**) UNet, **D**) CNN-GRU Model, **E**) CNN-LSTM Model

Figure 10 shows the simulation results based on learning curve of the all models for Scenario C which has the best results and the main approach for this study.

All five architectures show a downward trend in training loss, indicating that each model effectively learns from the dataset, yet they differ in the pace and stability of convergence. The 1D CNN and U-Net, for instance, exhibit rapid reductions in both training and validation loss, with consistently high and closely aligned accuracies, suggesting minimal overfitting and strong generalization. By contrast, the CNN-GRU and CNN-LSTM models reach high training accuracies but experience more pronounced fluctuations in validation accuracy, particularly due to their complex recurrent components. The Transformer balances learning capacity and robustness, with training and validation accuracies remaining close apart from occasional dips that recover over time, reflecting a capacity to adapt without severe overfitting. In 1D CNN, Transformer and UNet models, accuracies remain unchanged because the architectures quickly saturate on the available data, indicating that further epochs do not necessarily improve performance.

Overall, our proposed CNN-LSTM model has achieved notable success by effectively capturing both the spatial and temporal dynamics within the dataset. The model’s architecture—leveraging convolutional layers for feature extraction and LSTM layers for modeling temporal dependencies—has enabled it to achieve high training accuracy and competitive validation performance, despite some fluctuations that suggest a slight tendency towards overfitting. Importantly, this capability makes the CNN-LSTM model exceptionally well-suited for detecting the QE duration in archery, a critical factor in elite performance. By accurately identifying the QE phase, the model offers a promising tool for coaches and athletes to evaluate and refine their focus and performance strategies in real time. With further refinement through regularization techniques and hyperparameter tuning, the CNN-LSTM model holds significant potential to advance QE detection in archery, contributing to enhanced training protocols and overall competitive performance.

**Fig. 11:**
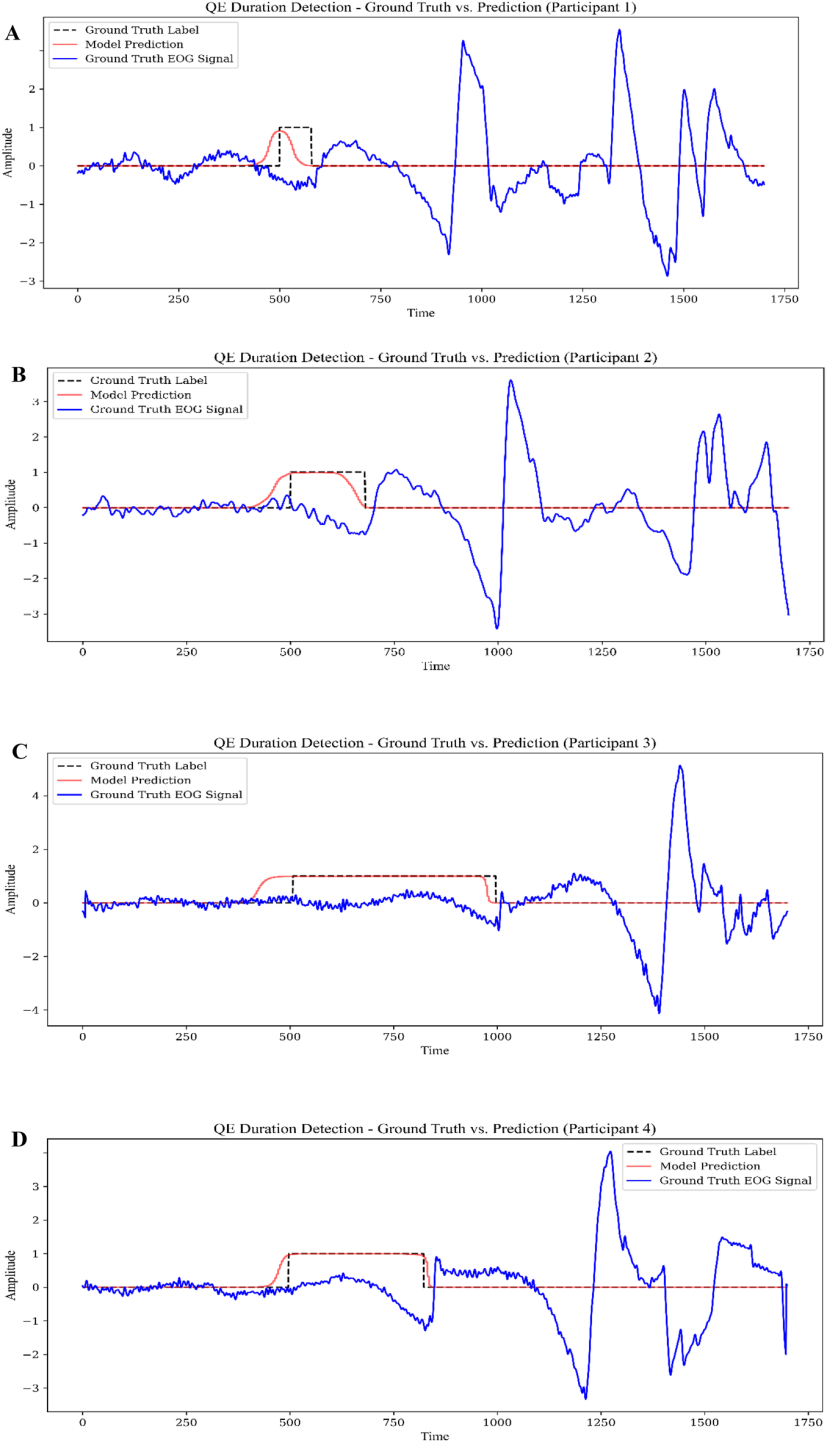
Sample predictions of the QE Durations using CNN-GRU hybrid model: (**A**)– (**D**), Participant 1– Participant 4

**Fig. 12:**
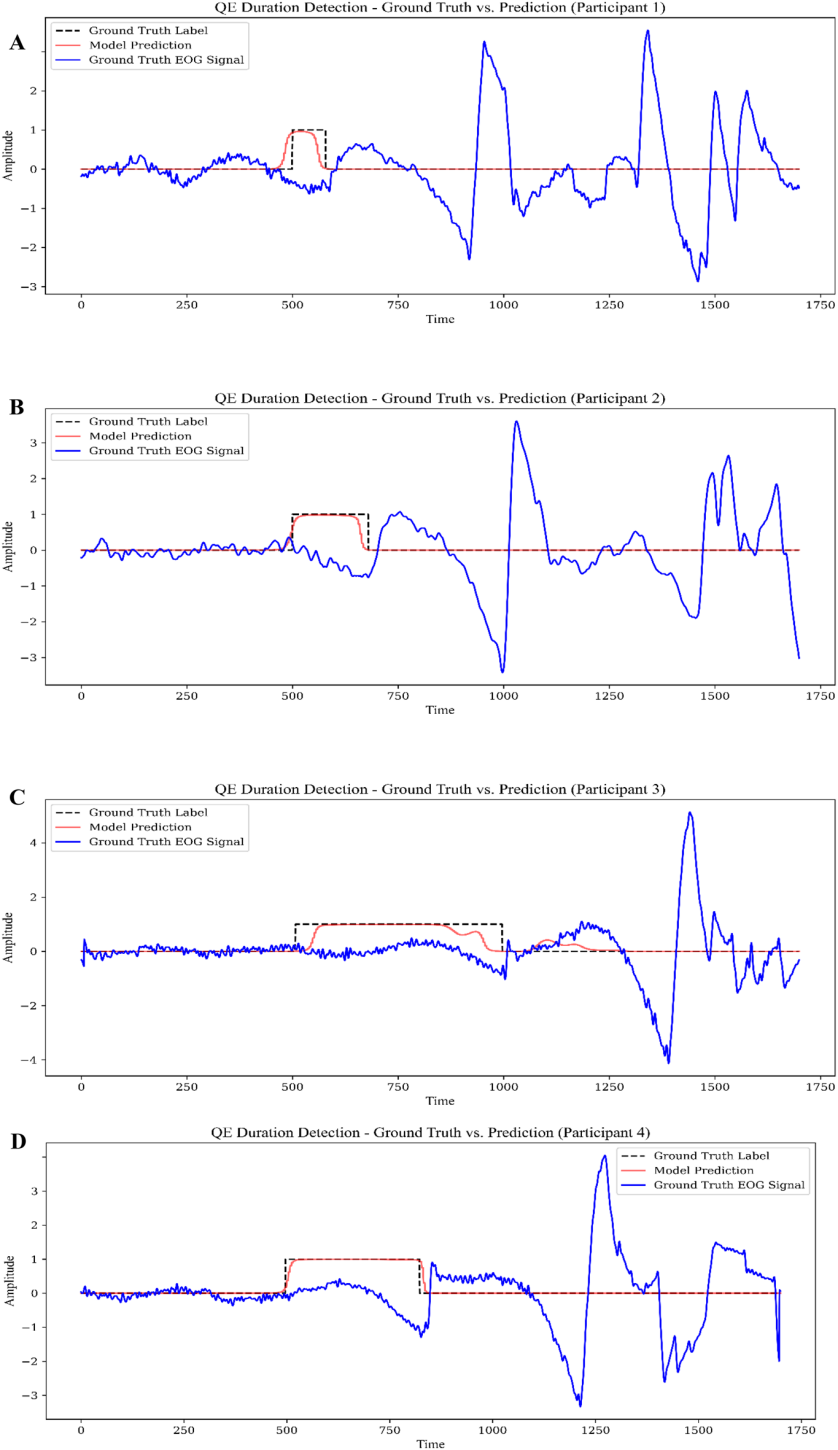
Sample predictions of the QE Durations using CNN-LSTM hybrid model: (**A**)– (**D**), Participant 1– Participant 4

Figure 11 shows sample predictions of QE durations with CNN-GRU hybrid model while Fig. 12 shows the results of CNN-LSTM model using same data.

Below is a detailed comparative discussion of the QE duration predictions made by the CNN-GRU and CNN-LSTM models for the same four participants since these two models have best performance metrics. Both approaches leverage convolutional layers for feature extraction and recurrent layers (GRU or LSTM) for temporal modeling, yet their predictions exhibit subtle differences in accuracy and interval alignment. Overall, both models capture the onset of QE events with reasonable precision, indicating that the convolutional front-end effectively learns spatial patterns in the EOG signal, while the recurrent component manages temporal dependencies. However, in several instances—particularly for Participants 2 and 4—the CNN-GRU model tends to slightly overextend the QE interval, whereas the CNN-LSTM model either aligns more closely with the ground truth or underestimates the offset by a smaller margin. For Participants 1 and 3, both models track the onset and offset times well, but the CNN-LSTM predictions appear marginally tighter around the labeled QE window, suggesting that the additional parameters and gating mechanisms of the LSTM might be refining how the model interprets subtle changes in the EOG signal. On the other hand, the CNN-GRU architecture displays more pronounced fluctuations in certain intervals, indicating a potential sensitivity to transient noise or signal spikes. Despite these nuances, both models demonstrate robust performance and underscore the feasibility of using deep learning to detect QE in archery. Ultimately, choosing between CNN-GRU and CNN-LSTM may hinge on domain-specific priorities—such as tolerance for slight over- or underestimation, computational resources, and the degree of temporal granularity required—yet both approaches provide a solid foundation for accurately identifying and analyzing QE events in real-world training scenarios.

In addition to the accuracy of each model, positive predictive value (PPV), sensitivity (SEN) and F1 score are also used as evaluation metrics for performance evaluation. These metrics are calculated from true positives (TP), false negatives (FN), and false positives (FP) by using the formulas below.

13 $$\:PPV=\frac{TP}{TP+FP}$$

14 $$\:SEN=\frac{TP}{TP+FN}$$

15 $$\:F1-score=2\:\times\:\frac{PPV\:\times\:SEN}{PPV+SEN}$$

PPV shows the number of correctly predicted values and SEN shows the number of correctly predicted positive cases, over all the positive cases in the data while F1-score measures the combination of both PPV and SEN. Finally, Mean Absolute Error (MAE) is calculated for the onset and offset predictions of QE events.

Performance metrics for each model is given in Table 2.

**Table 2:** Performance evaluation for each model for each scenarios

Scenario	Algorithm	Accuracy	PPV	SEN	F1-Score
A	SVM	~ 0.51	0.468 ± 0.012	0.473 ± 0.023	0.451 ± 0.076
	1D CNN	~ 0.77	0.753 ± 0.041	0.766 ± 0.004	0.751 ± 0.041
	Transformer	~ 0.75	0.736 ± 0.204	0.741 ± 0.331	0.758 ± 0.122
	UNet	~ 0.78	0.767 ± 0.010	0.782 ± 0.412	0.779 ± 0.255
	CNN-GRU	~ 0.82	0.819 ± 0.029	0.836 ± 0.204	0.842 ± 0.233
	**CNN-LSTM**	~ 0.84	0.856 ± 0.013	0.852 ± 0.323	0.851 ± 0.117
B	SVM	~ 0.64	0.632 ± 0.025	0.645 ± 0.031	0.628 ± 0.029
	1D CNN	~ 0.87	0.863 ± 0.019	0.875 ± 0.015	0.868 ± 0.017
	Transformer	~ 0.86	0.847 ± 0.094	0.855 ± 0.052	0.851 ± 0.067
	UNet	~ 0.87	0.865 ± 0.012	0.874 ± 0.028	0.869 ± 0.020
	CNN-GRU	~ 0.89	0.887 ± 0.035	0.899 ± 0.025	0.893 ± 0.029
	**CNN-LSTM**	~ 0.91	0.915 ± 0.014	0.922 ± 0.010	0.918 ± 0.012
C	SVM	~ 0.68	0.674 ± 0.298	0.692 ± 0.351	0.681 ± 0.022
	1D CNN	~ 0.90	0.844 ± 0.022	0.861 ± 0.013	0.852 ± 0.018
	Transformer	~ 0.89	0.829 ± 0.108	0.866 ± 0.037	0.847 ± 0.073
	UNet	~ 0.91	0.869 ± 0.009	0.883 ± 0.021	0.875 ± 0.015
	CNN-GRU	~ 0.92	0.914 ± 0.042	0.944 ± 0.019	0.928 ± 0.030
	**CNN-LSTM**	~ 0.93	0.948 ± 0.016	0.962 ± 0.006	0.954 ± 0.011

Table 2 presents the evaluation metrics, including PPV, SEN, and F1-Score, for the prediction of QE durations using different models across ten participants for each scenarios. These metrics serve as essential tools for assessing the performance of the models in capturing QE durations accurately.

Table 3 shows MAE for onset and offset of QE events for each models in different scenarios.

**Table 3:** Variability of MAE through all models for onset and offset prediction in each scenario

Onset-MAE(ms)						
Scenario	SVM	1D CNN	Transformer	UNet	CNN-GRU	**CNN-LSTM**
A	43	23	24	23	22	20
B	39	16	16	14	11	9
C	30	10	11	10	9	7

**Offset-MAE(ms)**						
Scenario	SVM	1D CNN	Transformer	UNet	CNN-GRU	**CNN-LSTM**
A	35	18	17	23	22	20
B	27	11	11	11	8	7
C	21	9	9	8	6	4

In general, filtered signal with baseline drift removal achieves the highest accuracies and lowest errors for each model. For each scenario, CNN-LSTM model achieves the highest accuracy which demonstrates a strong ability to predict QE durations, with high Precision values for most participants, indicating that the positive predictions made by the model are often accurate. Additionally, Recall values are generally high, signifying the model’s capability to correctly identify a substantial portion of the actual QE durations. This robust performance may be attributed to the LSTM’s gating mechanism, which captures temporal dependencies in the EOG signal without losing important context. The F1-Score, which strikes a balance between PPV and SEN, is also relatively high across the board. This suggests that the model achieves a favorable compromise between minimizing false positives and false negatives.

CNN-GRU achieves the second with an accuracy of ∼0.92, closely followed by UNet While both show strong metrics, CNN-GRU’s F1-score of 0.928 ± 0.030 indicates slightly greater stability in capturing true positives and limiting false positives compared to UNet. However, the UNet still demonstrates notable performance with an F1-score of 0.875 ± 0.015, likely due to its encoder-decoder structure, which can efficiently localize key features in the signal.

1D CNN and Transformer exhibit somewhat lower accuracy (∼0.90 and∼0.89, respectively), although they remain competitive. The 1D CNN benefits from its simplicity and lower computational overhead, making it a solid baseline with consistent performance (F1-score of 0.852 ± 0.018). The Transformer, while powerful in many sequence-to-sequence tasks, shows slightly higher variance in PPV (0.829 ± 0.108), possibly indicating that it is more sensitive to hyperparameter settings or requires a larger dataset to leverage its full capacity.

However, it’s worth noting that there is some variability in each model’s performance across participants. Some individuals exhibit near-perfect PPV and SEN, indicating exceptional predictive accuracy, while others have room for improvement. Several factors may contribute to these variations in model performance. Differences in shooting techniques, individual physiological factors affecting EOG signals, and variations in data quality may influence the model’s predictive accuracy. Further investigation into these participant-specific factors can help refine the model’s performance and address areas where improvements are needed.

MAE (Mean Absolute Error) metrics are a very useful tool for comparatively evaluating the ability of models to predict the onset and end timing of Quiet Eye (QE) events across different scenarios. In general, the CNN-LSTM model demonstrates its superiority in capturing temporal dependencies by achieving the lowest MAE values ​​in both metrics (Onset and Offset) across all scenarios. This success is based on the fact that the LSTM’s gate mechanism effectively models time-varying patterns in EOG signals. The CNN-GRU model also demonstrates how suitable temporal models are for such tasks by achieving a performance very close to CNN-LSTM. On the other hand, the SVM model consistently achieves the highest MAE values ​​across all scenarios, indicating that it offers limited success in time series-based prediction tasks especially for non-stationary signals.

Among the scenarios, in Scenario C models achieved the lowest MAE values in prediction. This suggests that less noise and more quality in signals may improve the timing accuracy of the models. In contrast, Scenario A presents the highest MAE values, implying that this scenario is more challenging. Additionally, small differences were observed between the initial and final estimates for some models (e.g., 1D CNN and Transformer), reflecting the varying sensitivity of the models to different timing events.

As a result, MAE metrics provide a critical measure for assessing how accurately models can predict the timing of QE periods. While deep learning models such as CNN-LSTM and CNN-GRU clearly outperform others in this task, SVM, as a traditional approach falls short in such complex time series analyses.

The statistical analyses show that temporal models such as CNN-LSTM and CNN-GRU perform statistically significantly better than traditional models such as SVM in all scenarios. In particular, CNN-LSTM proved to be the most effective model in the task of predicting the timing of QE events, outperforming other models in both accuracy and MAE metrics. However, the differences between CNN-LSTM and CNN-GRU were not found to be statistically significant in some scenarios, suggesting that both models have similar success in modeling temporal dependencies. Future work can focus on additional optimizations or hybrid approaches to further improve the performance of these models.

Automating the determination of QE in archery yields valuable insights; however, the system has several limitations. This study utilized data from ten archers, and EOG signals may differ among archers of varying age groups and skill levels (e.g., amateur vs. olympic-level) due to physiological variations. Future studies should expand to larger, more diverse populations, potentially across different archery disciplines, to confirm the generalizability of these findings. Moreover, exploring individual differences in EOG signal profiles could yield deeper insights into how physiological and skill-related factors influence QE duration and, consequently, shooting performance. Consequently, when applied to a broader population, the system may exhibit reduced accuracy and may necessitate extensive recalibration or retraining. Also, the method employed in this study operates in an offline setting. Nevertheless, for active archers, immediate feedback on QE duration is essential. Given these limitations, future research should involve larger and more diverse participant samples to enable more generalizable and effective applications.

In summary, the ranking of the models in terms of average performance across participants is led by the CNN-LSTM and CNN-GRU, followed by UNet, 1D CNN, and Transformer. The recurrent-based architectures (CNN-LSTM and CNN-GRU) seem to excel at handling the temporal aspects of QE detection, while UNet stands out for its strong performance in extracting spatial features. The simpler 1D CNN and the Transformer remain viable options, particularly if computational or architectural constraints are a consideration.

## Conclusion

In this study, we extended our previous work by systematically comparing one traditional machine learning algorithm SVM and five distinct deep learning models—namely 1D CNN, Transformer, UNet, CNN-GRU, and CNN-LSTM—for the automated detection of QE durations from EOG signals. In the study, the evaluation metrics of EOG signals with different signal qualities were compared as input to the model, and thus the study became a very comprehensive study that will contribute to the relevant literature. Our results demonstrate that while all models effectively capture the essential spatial and temporal characteristics of EOG data, the hybrid recurrent architectures exhibit superior performance. The study has become very comprehensive with the results obtained by giving inputs with different signal qualities to artificial intelligence.Specifically, the CNN-LSTM model achieved the highest accuracy (∼93%) along with robust precision (PPV = 0.948 ± 0.016), sensitivity (SEN = 0.962 ± 0.006), and F1-score (0.954 ± 0.011), thereby offering a significant improvement over traditional expert-based evaluations and other deep learning alternatives.

The CNN-GRU model closely follows, with an accuracy of approximately 92% and an F1-score of 0.928 ± 0.030, reflecting its effective balance between capturing temporal dependencies and managing computational efficiency. The UNet model, benefiting from its encoder-decoder architecture, achieved commendable results as well, while the simpler 1D CNN and the Transformer models, despite exhibiting competitive performance (accuracy ∼0.90 and ∼0.89, respectively), displayed slightly lower metrics. Notably, the Transformer model’s higher variance in precision suggests a sensitivity to hyperparameter tuning and potential benefits from larger datasets.

These findings not only validate the importance of QE as a performance indicator in precision sports like archery but also underscore the potential of advanced machine learning techniques to provide objective, automated measurements that overcome the limitations of subjective manual annotations. By accurately detecting QE durations, our approach can inform targeted training interventions and real-time performance assessments, with broader applications in domains requiring high levels of visual focus and precision, such as military and law enforcement training.

While our current investigation, based on data from 10 archers, offers promising insights, future research should focus on larger, more diverse populations to enhance generalizability and further refine model performance. Additionally, investigating individual differences in EOG signal characteristics may yield deeper understanding of the factors influencing QE duration and overall athletic performance. The findings underscore the potential of our hybrid approach to quantify the relationship between athlete performance and QE, particularly in precision sports. By automating the detection process, this study lays the groundwork for future research exploring the early identification of performance indicators and the effects of targeted training interventions on athletic outcomes. Furthermore, the applicability of this method extends beyond athletics; it holds promise for assessing targeting skills in other fields, such as military and law enforcement, where visual focus and precision are critical.

While the methodology has been validated in an offline setting, it is important to note that this is a preliminary study. Future work aims to implement real-time QE detection systems, which would enhance training and performance assessment in dynamic environments. Overall, this study contributes to the understanding of QE in sports science and offers a scalable solution for its measurement, paving the way for further exploration in related domains.

In summary, the present study contributes to the growing literature on QE by demonstrating that a hybrid CNN-LSTM model, and to a slightly lesser extent, the CNN-GRU model, offer effective and scalable solutions for automated QE detection. This advancement lays the groundwork for real-time applications and further exploration of performance indicators in sports science and beyond.

## Data Availability

The datasets used and/or analyzed during the current study are available from the corresponding author on reasonable request.

## Abbreviations

QE: Quiet Eye
EOG: Electrooculography
ML: Machine Learning
DL: Deep learning
SVM: Support Vector Machines
CNNs: Convolutional Neural Networks
RNNs: Recurrent Neural Networks
LSTM: Long Short-Term Memory
ReLU: Rectified Linear Unit
GRU: Gated Recurrent Units
PPV: Positive predictive value
SEN: Sensitivity
TP: True positives
FN: False negatives
FP: False positives
MAE: Mean Absolute Error
